# Isolation and Evaluation of Mucilage of *Adansonia digitata* Linn as a Suspending Agent

**DOI:** 10.1155/2013/379750

**Published:** 2013-12-24

**Authors:** S. S. Deshmukh, Y. S. Katare, S. S. Shyale, S. S. Bhujbal, S. D. Kadam, D. A. Landge, D. V. Shah, J. B. Pawar

**Affiliations:** ^1^Hon.Shri Babanrao Pachpute Vichardhara Trust's GOI, College of Pharmacy, Kashti, Tal-Shrigonda, Ahmednagar District, Maharashtra 414701, India; ^2^Padmashree Dr. D. Y. Patil Institute of Pharmaceutical Sciences & Research, Pimpri, Pune, 411018, India

## Abstract

Natural excipients can serve as alternative to synthetic products because of local accessibility, biodegradability, eco-friendly nature and cost effectiveness as compared to synthetic products. Therefore, it is a current need to explore natural excipients that can be used as an effective alternative excipient for the formulation of pharmaceutical dosage forms. *Adansonia digitata* (Malvaceae) has been traditionally used as febrifuge, antiasthmatic and also in the treatment of dysentery, smallpox, and measles. Reports have indicated that mucilage of the leaves of the plant is edible and nontoxic; hence, the present study is an attempt of isolation and evaluation of mucilage obtained from leaves of *Adansonia digitata* as suspending agent. Various physicochemical as well as suspending agent properties of mucilage were studied. Mucilage obtained from leaves has shown comparable results with sodium carboxy methyl cellulose.

## 1. Introduction

Natural polymers have been used in different pharmaceutical formulations. They are easily available, nontoxic, biodegradable, and cost effective to be used as pharmaceutical excipients [1, 2].

In recent years, plant-derived polymers such as mucilages can occur in high amounts in different parts of the plant and have evoked tremendous interest due to their diverse pharmaceutical applications such as diluent, binder, disintegrant in tablets, thickeners in oral liquids, protective colloids in suspensions, gelling agents in gels, and bases in suppositories. They are also used in cosmetics, textiles, paints, and paper making. These hydrocolloid natural gums and mucilage are biocompatible, cheap, and easily available and are preferred to semisynthetic and synthetic excipients because of their lack of toxicity, low cost, easy availability, soothing action, and nonirritant nature. Demand for these substances is increasing, and new sources are being developed. India, because of its strategic location, geographically and environmentally, has been traditionally a good source for such products amongst the Asian countries [3].

Mucilage is a water soluble, sticky, and gummy substance obtained from certain plants. In plants, it acts as a membrane thickener and food reserve. Gums swell in water to form slippery and aqueous colloidal dispersions. Mucilage occurs in nearly all classes of plants in various parts of the plant, including marsh mallows, flaxes, and certain seaweeds in relatively small percentages and other substances such as tannins and alkaloids are also occasionally found [4]. Gums are widely employed in the pharmacy as thickeners, suspending agents, emulsifying agents, binders, and film formers. With the increase in demand for natural gums, it has been necessary to explore the newer sources of gums to meet the industrial demands [5].

A pharmaceutical suspension, like other disperse systems, is thermodynamically unstable, thus making it necessary to include in the dosage form a stabilizer or suspending agent which reduces the rate of settling and permits easy redispersion of any settled particulate matter both by protective colloidal action and by increasing the consistency of the suspending medium. Suspending agents are (i) inorganic materials, (ii) synthetic compounds, or (iii) polysaccharides [6].

The *Adansonia digitata* tree occurs in many parts of the tropics, mainly as a component of secondary forest. Only the leaves are mucilaginous and are consumed fresh or dried. Soups prepared from the leaves are particularly popular as a weaning food. 10% w/w of the dry matter of the leaves contains mucilage and the mucilage is also found to be acidic polysaccharide with proteins and minerals [7].

Hence, the present study is an humble effort to isolate mucilage from *Adansonia digitata* leaves and to evaluate its feasibility as suspending agent for suspensions.

## 2. Materials and Methods

### 2.1. Collection of Plant Material

The Leaves of *Adansonia digitata* were collected from Ahmednagar, Maharashtra, India, and authenticated by Senior Botanist, Department of Botany, MJS College, Maharashtra, India.

### 2.2. Isolation of Mucilage

The leaves of *Adansonia digitata* were dried, powdered, and sieved through number 120. 100 gm of the undersized powder particles was mixed with 500 mL of distilled water and allowed to settle for 24 h. The mixture was boiled for 1 h at 100°C and kept aside for 2 h for settling. After 2 h, suspension was filtered, and to the filtrate equal volume of ethanol was added and kept in refrigerator at 8–10°C for 24 h. Precipitate obtained was separated by filtration through muslin fabric and the residue over the filter bed was collected and stored separately in a clean, dry, and closed container [8].

### 2.3. Preparation of Paracetamol Suspensions

Compound sodium carboxy methyl cellulose (CMC) powder (0.25 g) and 10 g of paracetamol were triturated together with 20 mL of raspberry syrup to form a smooth paste. Benzoic acid solution (2 mL) and 1 mL of amaranth solution were added gradually with constant stirring and then mixed with 50 mL of chloroform water (double strength). The mixture was transferred into a 100 mL amber coloured, stoppered measuring cylinder, made up to volume with distilled water and then shaken vigorously for 2 min (thus making 0.25% w/v of the preparation). The procedure was repeated using 0.5% w/v and 1.0% w/v of Sodium CMC powder. The above procedure was repeated with mucilage isolated from leaves of *Adansonia digitata *at concentrations 0.25% w/v, 0.5% w/v, and 1.0% w/v [5].

### 2.4. Evaluation of Suspensions

#### 2.4.1. Phytochemical Tests of Isolated Mucilage

Phytochemical tests for the presence of carbohydrates, glycosides, proteins, flavonoids, alkaloids, and tannins in the mucilage were performed as per standard procedure [9, 10].

#### 2.4.2. Physicochemical Characteristics of Isolated Mucilage

Physicochemical characteristics of isolated mucilage such as colour, odour, taste, nature, solubility, ash value, acid insoluble ash value, water soluble ash value, loss on drying, and swelling index were performed as per procedure [9, 10].

*(A) Physical Test. *For a period of 4 weeks, everyday, the prepared suspensions were observed for physical changes such as aggregation, caking, and crystal growth formation [11].

*(B) Redispersibility. *Fixed volume (50 mL) of each suspension was kept in separate calibrated tubes and stored at room temperature. At regular intervals of 24 h, a tube was shaken to observe ease of redispersibility of the sediment and also for the presence of deposits. The observations were recorded [11].

*(C) Determination of Sedimentation Volume. *Each suspension (50 mL) was stored in a 50 mL amber coloured, stoppered measuring cylinder for 4 days at 35°C, and observations were made every 24 h for 4 days. The sedimentation volume, *F* (%), was then calculated using the following equation. Triplicate readings were obtained and average was computed [12]:

(1) $$\begin{array}{c} F=\frac{100V_{u}}{V_{o}}, \end{array}$$

where *V*_*u*_ is volume of the sediment and *V*_*o*_ is total volume of suspension.

*(D) Measurement of Viscosity Using the Brookefield Viscometer.* The viscosity of the sample was determined at 25°C using the Brookefield synchroelectric viscometer, model RVDV-PRO II (Brookfield, USA) at 100 RPM (spindle number 4). All determinations were made in triplicate and the results obtained were expressed as the mean values [12].

*(E) Determination of Flow Rate. *The time required, for a fixed volume of suspension, to flow through a orifice, for a fixed distance, was determined. Average of 3 readings was consistently recorded [12]:

(2) $$\begin{array}{c} \text{flow}\;\text{rate}=\frac{\text{distance}\;\left(\text{cms}\right)}{\text{flow}\;\text{time}\;\left(\mathrm{Min}\right)}. \end{array}$$

## 3. Results

The mucilage obtained by the above outlined procedure was subjected to phytochemical tests. The phytochemical tests revealed that mucilage was present in adequate amounts (Ruthenium test). Further, the tests also revealed the presence of carbohydrates and absence of tannins, proteins, alkaloids, glycosides, and flavonoids. Results of these tests are summarised in Table 1.

Physicochemical characterization of isolated mucilage showed that it is green in colour having mucilaginous taste and characteristic odour. It is soluble in water but insoluble in ethanol and chloroform. The mucilage was also examined for its total ash value, acid insoluble ash, water of soluble ash, loss on drying, and swelling index, and the results obtained are summarized in Table 2.

The paracetamol suspensions (500 mg/5 mL) were prepared the using isolated mucilage at different levels, that is, 0.25%, 0.5%, and 1% w/v, and similar suspensions were prepared with sodium CMC. These suspensions were assessed for their redispersibility, sedimentation volume, viscosity, and flow rate and the results were compared.

When the suspensions were observed during the first 48 h, no aggregation of particles, or caking or crystal growth formation, was observed.

The suspensions were shaken at the end of 24 h for several days to assess redispersibility of paracetamol. It was observed that suspensions containing mucilage and sodium CMC at 0.25% and 0.5% showed quick settling of particles. However, suspensions containing mucilage or sodium CMC at 1% concentration showed gradual and slow settling of particles. The results are in conformation with the flow rate study.

**Figure 1:**
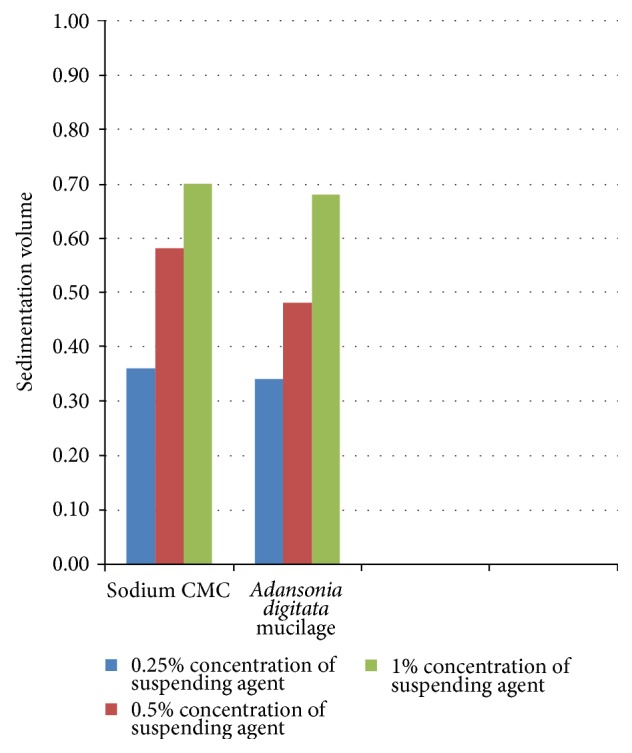
Determination of sedimentation volume (*F*).

**Figure 2:**
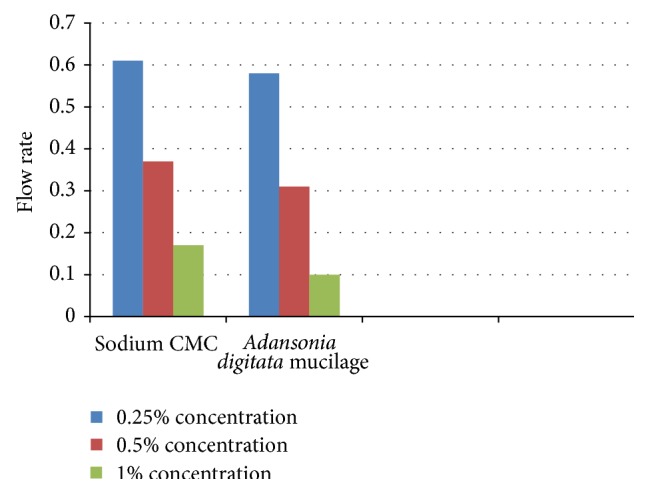
Determination of flow rate of suspension.

Sedimentation volume was determined as percentage and is depicted in Figure 1. The study shows that increasing the amounts of suspending agents either mucilage or sodium CMC shows increase in the percentage of sedimentation volume. Similarly, when the suspensions were studied for their rheology, the viscosity of mucilage containing suspensions showed 1.05, 1.58, and 2.10 poises, respectively, for 0.25% 0.5%, and 1% mucilage containing suspensions. Similarly, for Sodium CMC containing suspensions, viscosities observed were, 0.57, 0.70, and 0.85 poises, respectively, for 0.25% 0.5%, and 1% of Sodium CMC (see Figures 1 and 2). The results are tabulated in Table 3.

## 4. Discussion

The *Adansonia digitata* shows many medicinal properties and also contains many phytoconstituents; the leaves contains 7–10% mucilage [7]. It was understood from the literature survey that the natural mucilage can be used as a pharmaceutical adjuvant, and further there was minimal or no work carried out to explore its feasibility as a suspending agent. Therefore, in this study, the suspending ability of the isolated mucilage was assessed.

Phytochemical tests were carried out on mucilage which confirmed the presence of carbohydrates and mucilage. It was also found that proteins, alkaloids, glycosides, tannins, and flavonoids were absent. Therefore from this study, it was inferred that the mucilage obtained from *Adansonia digitata* is of excellent quality since it remains uncontaminated with other substances or chemicals.

Suspensions tend to form sediment on storage, and, hence they must be readily dispersible so as to ensure the uniformity of the dose. If sediment remains even after shaking vigorously for specified time, the system is described as caked [5]. All the six formulations of either mucilage or sodium CMC were found to be easily redispersible.

Suspensions are the least stable dosage form due to sedimentation and cake formation. As the viscosity of the suspension increases, the terminal settling velocity decreases; thus, the dispersed phase settles at a slower rate and remains dispersed for a longer time yielding higher stability to the formulated suspension. The less viscous suspension tends to pour more easily than the more viscous ones, and hence study of rheology or viscosity is critical to understand stability of suspensions [11]. In this work, viscosities of suspensions were studied in Brookefield viscometer and the results were tabulated in Table 3. The results indicate that as the amounts of the suspending agent increases, viscosity also increases gradually. The viscosities of isolated mucilage and sodium CMC are comparable.

Similarly, the flow rates of the suspensions were determined by standard method as discussed in methods section. The results showed that as the concentration of mucilage or sodium CMC increases, viscosity increases and consequently flow rate decreases gradually.

The results obtained so far therefore have indicated that, the mucilage isolated from *Adansonia digitata* has the potential to be used as a suspending agent; however, its actual *in vivo* performance on suitable animals or humans remains to be studied.

## 5. Conclusions

*Adansonia digitata* is a potential plant useful in the treatment of various diseases, and, in this study, it is concluded that the isolated mucilage, which contains mainly carbohydrates, can also be used as a suspending agent. However, its potentiality to be used as a tablet adjuvant is being explored in our laboratories.

## Figures and Tables

**Table 1:** Phytochemical screening of mucilage of *Adansonia digitata. *

Sr. no.	Tests	Observations
1	Test for carbohydrates (Molisch's test)	Positive
2	Test for tannins (Ferric chloride test)	Negative
3	Test for proteins (Ninhydrin test)	Negative
4	Test for alkaloids (Wagner's test)	Negative
5	Test for glycosides (Keller-Killaini test)	Negative
6	Test for mucilage (Ruthenium red test)	Positive
7	Test for flavonoids (Shinoda test)	Negative

**Table 2:** Physicochemical screening of mucilage of *Adansonia digitata.*

Sr. no.	Evaluation properties	Observations
1	Colour	Light green
2	Odour	Characteristics
3	Taste	Mucilaginous
4	Nature	Crystalline
5	Solubility	Forms colloidal solution in water and insoluble in alcohol and chloroform
6	Total ash value	3.44%
7	Acid insoluble ash value	1.5%
8	Water soluble ash value	1.88%
11	Loss on drying	11.8%
13	Swelling index	3

**Table 3:** Effect of type and concentration of suspending agent on viscosity.

Suspending agent	Concentration % w/v	Viscosity (poise)
*Adansonia digitata* mucilage	0.25	1.05
	0.5	1.58
	1	2.10

Sodium CMC	0.25	0.57
	0.5	0.70
	1	0.85
